# Oncolytic vaccinia virus expressing a bispecific T-cell engager enhances immune responses in EpCAM positive solid tumors

**DOI:** 10.3389/fimmu.2022.1017574

**Published:** 2022-11-14

**Authors:** Min Wei, Shuguang Zuo, Zhimin Chen, Peng Qian, Yenan Zhang, Lingkai Kong, Honglan Gao, Jiwu Wei, Jie Dong

**Affiliations:** ^1^ Affiliated Yancheng No.1 People’s Hospital, Medical School of Nanjing University, Yancheng, China; ^2^ Jiangsu Key Laboratory of Molecular Medicine, Medical School of Nanjing University, Nanjing, China; ^3^ Liuzhou Key Laboratory of Molecular Diagnosis, Guangxi Key Laboratory of Molecular Diagnosis and Application, Affiliated Liutie Central Hospital of Guangxi Medical University, Liuzhou, Guangxi, China

**Keywords:** vaccinia virus, bispecific T-cell engager, cancer therapy, EpCAM, solid tumor

## Abstract

Insufficient intratumoral T-cell infiltration and lack of tumor-specific immune surveillance in tumor microenvironment (TME) hinder the progression of cancer immunotherapy. In this study, we explored a recombinant vaccinia virus encoding an EpCAM BiTE (VV-EpCAM BiTE) to modulate the immune suppressive microenvironment to enhance antitumor immunity in several solid tumors. VV-EpCAM BiTE effectively infected, replicated and lysed malignant cells. The EpCAM BiTE secreted from infected malignants effectively mediated the binding of EpCAM-positive tumor cells and CD3ϵ on T cells, which led to activation of naive T-cell and the release of cytokines, such as IFN-γ and IL-2. Intratumoral administration of VV-EpCAM BiTE significantly enhanced antitumor activity in malignancies with high other than with low EpCAM expression level. In addition, immune cell infiltration was significantly increased in TME upon VV-EpCAM BiTE treatment, CD8^+^ T cell exhaustion was reduced and T-cell-mediated immune activation was markedly enhanced. Taken together, VV-EpCAM BiTE sophistically combines the antitumor advantages of bispecific antibodies and oncolytic viruses, which provides preclinical evidence for the therapeutic potential of VV-EpCAM BiTE.

## Introduction

Over the past few years, cancer immunotherapy has achieved prominent therapeutic success (1, 2). Among the immunotherapy modalities, oncolytic viruses may provide a simple and *in situ* inoculation method to activate T-cell responses by inducing immunogenic tumor cell death, releasing mutant neoantigens and altering the immunosuppressive tumor microenvironment (3, 4). Several oncolytic viruses, such as vaccinia virus (VV) and herpes simplex virus (HSV), have been used in solid tumor immunotherapy studies (5–8). In particular, recombinant vaccinia viruses have shown exciting results. JX594, a vaccinia virus arming granulocyte macrophage colony-stimulating factor (GM-CSF), has proven to be effective in global phase I/II clinical trials for patients with hepatocellular liver cancer (6, 9). TG6002, another vaccinia virus expressing a suicide gene with deletion of TK and RR genes, displayed antitumor activity in preclinical research (10, 11). The combination of TG6002 with 5-fluorocytosine is ongoing in phase I/II clinical trials for the treatment of gastrointestinal cancers (NCT03724071 and NCT04194034) and recurrent glioblastoma (NCT03294486) (12). Due to its ability to carry a large variety of exogenous genes and infect broad host cells, vaccinia virus has emerged as one of the most promising oncolytic viral candidates for clinical use (13–15).

Bispecific T-cell engager (BiTE) is a novel immunotherapeutic protein consisting of two single chain variable fragments (scFvs) connected *via* a flexible linker that target both CD3 and tumor-associated antigens (16). BiTE builds a bridge between cytotoxic T cells and tumor cells, subsequently leading to T-cell activation and specific tumor cell lysis (17). Transient release of cytokines engages other immune cells and amplifies the immune responses against tumors (18, 19). Blinatumomab, a BiTE targeting CD19, was approved by the FDA for the treatment of acute lymphoblastic leukemia, and this established that the principle of polyclonal T-cell engagement with tumor targets is feasible and effective (20). Nevertheless, the success of BiTE therapy in hematologic malignancies has not been replicated in solid tumors until now. Many efforts have been made in BiTEs targeting different solid tumor-associated antigens, including the epithelial cell adhesion molecules (EpCAM), human epithelial growth factor receptor-2 (HER2), epidermal growth factor receptor (EGFR), carcinoembryonic antigen (CEA), Ephrin type-A receptor 2 (EphA2) and prostate stem cell antigen (PSCA) (18, 21–25). However, due to poor clinical responses and several side effects, such as dose-limiting toxicity, “on-target, off-tumor” toxicity, cytokine release syndrome and central nervous system (CNS) events, no BiTEs are yet marketed for solid tumors (26–28).

To address the problems above, oncolytic viruses are used as vectors for tumor targeting, and the BiTE-encoding oncolytic virus (OV-BiTE) platform has the potential to overcome these limitations. The first study illustrating the functionality of an oncolytic virus encoding BiTE was by Yu et al. in 2014; they studied an EphA2-targeted T-cell engager arming vaccinia virus named EphA2-TEA-VV. In the A549 model, EphA2-TEA-VV treatment significantly inhibited tumor growth (29). In this study, we also generated an engineered oncolytic vaccinia virus, VV-EpCAM BiTE, which expresses and secretes a soluble EpCAM-targeted T-cell engager. Herein, we systematically investigated the antitumor efficacy of this recombinant virus in different solid tumor models and found that VV-EpCAM BiTE results in superior tumor suppression effects compared to VV-Ctrl in EpCAM-positive tumor cells. Our study provides a novel recombinant oncolytic virus for the effective treatment of solid tumors and strong preclinical evidence for the feasibility of OV-BiTEs.

## Materials and methods

### Cell lines

Human embryonic kidney cell HEK293, mouse mammary carcinoma cell 4T1, mouse colon carcinoma cell CT26, human cervix carcinoma cell HeLa-S3, mouse melanoma cell line B16F10, Vero and BEAS-2B cells were obtained from the American Type Culture Collection (ATCC, USA). Mouse colon carcinoma cell line MC38 was purchased from the National Cancer Institute (NCI; USA). The mouse HCC cell line H22 was purchased from the China Center for Type Culture Collection (CCTCC; Wuhan, China). H22 cell was cultured in RPMI 1640 medium (Cat#11875093, Gibco) supplemented with 10% fetal bovine serum (FBS, Cat#16000044, Gibco) and other cell lines were cultured in DMEM supplemented with 10% FBS. All cells were maintained in a humidified incubator with an atmosphere containing 5% CO_2_ at 37°C.

### Preparation of recombinant vaccinia viruses

The pSEL shuttle plasmids encoding EpCAM-scFv-CD3-scFv-GFP, CD19-scFv-CD3-scFv-GFP or GFP were constructed. The inserted chimeric genes were under the control of the VV p7.5k promoter. Then the shuttle plasmids were transfected into HEK293 cells together with the infection of vaccinia virus western reserve strain so that the shuttle plasmids insert genes of interest into the TK locus of VV. Then the recombinant viruses were picked by plague screening to obtain the recombinant VV expressing EpCAM-scFv-CD3-scFv (VV-EpCAM BiTE), CD19-scFv-CD3-scFv (VV-Ctrl-BiTE) or GFP (VV-Ctrl). The recombinant viruses were then amplified in HeLa S3 cells and purified using sucrose gradient ultracentrifugation. Virus titration was determined by adding serially diluted virus into a 96-well plate seeded with HEK293 cells (10000 cells/well). Cells were cultured for 4 days, and fluorescence was evaluated *via* microscopy. The virus titer was calculated according to the following formula:


$$\text{TCID}50=10^{2+\left(\text{S}/\text{N}-0.5\right)}/\text{mL}$$

PFU/mL=0.7×TCID50/mL

where S is the total number of fluorescence-positive wells, and N is the number of replicates.

### Viral oncolysis

The *in vitro* oncolysis assay was measured by MTT. 4T1, B16F10, CT26, MC38, Vero, BEAS-2B, LO2 cells were seeded in a 96-well plate at 1 x 10^4^ cells per well and recombinant oncolytic VVs were added at the indicated MOI. After a 72 h incubation, 20 μl of MTT solution (5 mg/ml; Cat# IM0280, Solarbio, Beijing, China) was added to each well and the cells were incubated for 4 h. The supernatants were discarded and 150 μl DMSO was added to each well. The absorbance (A) was tested at a wavelength of 570 nm using microplate reader (Spectra-Max M3, Molecular Devices, USA). The cell viability was calculated according to the following formula:


$$\text{Cell viability}\left(\%\right)=\left({\text{A}}_{\text{treatment}}-{\text{A}}_{\text{blank}}\right)/\left({\text{A}}_{\text{control}}-{\text{A}}_{\text{blank}}\right)\times 100\%$$

### Viral replication

Viral replication was determined both *in vitro* and *in vivo*. *In vitro,* cells were seeded in a 24-well plate at 1 x 10^5^ cells per well and infected with recombinant oncolytic VVs at an MOI of 0.1. The cells were harvested at indicated time interval and lysed by three freeze-thaw cycles. The virus supernatants were collected and virus titers were determined by a TCID50 method. *In vivo,* whole blood and tumor tissue were collected from mice on day 3 and 7 after VV treatment. Tumor samples were then lysed with a homogenizer and then the supernatants were harvested. Serum was separated from the whole blood by centrifugation. Then either supernatants of tissue lysates or sera were determined by a TCID50 method.

A 4T1 mouse subcutaneous tumor model was established and VV-EpCAM BiTE 1 x 10^7^pfu was injected intratumorally. 48 hours after virus treatment, organs or tissues including heart, liver, spleen, lung, kidney, brain, small intestine and muscle were taken from the mice. RNA was extracted, RT-PCR was performed and DNA templates were prepared. Primers designed based on the epidermal growth factor-like (C11R) gene secreted by VV (forward, 5’-AAACACACACTGAGAAACAGCATAAA-3’; reverse, 5’-ACTCGGCGAATGATCTGATTA-3’). Subsequently, the standard quantitative PCR was performed on the prepared DNA template using AceQ Universal SYBR qPCR Master Mix (Cat# Q511, Vazyme, Nanjing, China).

### Animal experiments

Six-week-old C57BL/6J and BALB/c mice were purchased from Nanjing University Model Animal Institute (Nanjing, China). All animal were fed in a specific pathogen-free (SPF) grade environment and experiments were performed in accordance with the guidelines approved by the Animal Care and Use Committee of the Medical School of Nanjing University. For the solid tumor models, 4T1, MC38-EpCAM, MC38, or H22 tumor cells were injected subcutaneously into the right flank of the mice. When the tumor volumes reached approximately 100 mm^3^, the mice were randomly divided into different groups. Each mouse was intratumorally administered with 10^7^ plaque-forming units (PFUs) of VV three times, and injected with PBS as a control. Tumor volume and body weight were measured every other day. Tumor volume (V) was calculated by the following formula: V = (length × width^2^)/2. Mice were sacrificed when the volume reached 2.0 cm^3^. For the 4T1 model, when the subcutaneous tumor volume was approximately 100 mm^3^, we also performed tail vein injections of VV-EpCAM BiTE at 1x10^7^ pfu each time, on alternate days, for a total of three treatments.

### Western blot

HEK293 cells were seeded in six-well plates of 5x10^5^ cells per well. After the cells are completely attached, HEK293 cells were infected with VV-Ctrl-BiTE or VV-EpCAM BiTE at the MOI of 1. After 48 hours, the supernatants were harvested and centrifuged to remove cells or cellular debris. The primary antibody was anti-6x-His tag monoclonal antibody (Cat# MA1-21315, Invitrogen). The secondary antibody was Goat anti-Mouse IgG (H+L) (Cat# A16068, Invitrogen).

### Enzyme-linked immunosorbent assay

Splenocytes isolated from BALB/c female mice or C57BL/6J male mice were cocultured with 4T1, H22, MC38-EpCAM and MC38 cells containing luciferase at effector: target (E:T) ratio of 10:1 and subsequently, supernatants containing BiTE were added to the coculture system. After 48h, the supernatants were harvested and the levels of IL-2 and IFNγ cytokines were quantified by ELISA kits according to the manufacturer’s instructions (Biolegend, USA).

### Flow cytometry

To detect the expression of EpCAM on the cell surface, cultured 4T1, MC38, MC38-EpCAM and H22 cells were harvested and stained with monoclonal antibodies against mouse EpCAM/CD326 (PE; Clone G8.8, Cat# 12-5791-82, eBioscience, USA) for 15 min at room temperature in the dark. For the binding assay, we established a coincubation system, in which EpCAM-positive 4T1 cells were coincubated with EpCAM BiTE or control BiTE-containing supernatants at room temperature for half an hour. Afterwards, the supernatants were discarded by centrifugation and washed with PBS, stained with antibodies against mouse EpCAM for 15 min in the dark and immediately detected by flow cytometry.

For *in vitro* T cell activation assay, a single suspension from mice spleen was prepared using 70 μm strainer. The cells were then centrifuged at 300g for 10 min, resuspended in erythrocyte lysate, left to stand at 4°C for 5 min, centrifuged at 300g for 10 min, washed again with 1x PBS, and finally resuspended in 1x PBS. 4T1, H22, MC38-EpCAM and MC38 cells were seeded in 12-well plates with 1 x 10^5^ cells per well. Splenocytes were added to the 12-well plates according to an effector to target (E:T) ratio of 10:1, followed by co-incubation with 1 ml of supernatant containing BiTE. After 48 hours, the cells were stained for CD25-APC (Clone PC61, Cat# 557192, BD), CD45 (PerCP/Cyanine5.5, Clone 30-F11, Cat# 103129, BioLegend; APC, Clone 30-F11, Cat# 559864, BD), CD4-FITC (Clone GK1.5, Cat# 100405, BioLegend), CD8a-PerCP/Cyanine5.5(Clone 53-6.7, Cat# 100734, BioLegend), CD69-FITC (Clone H1.2F3, Cat# 104505, BioLegend) and CD3-PE (Clone 17A2, Cat# 100206, BioLegend) and analyzed by flow cytometry. The cytotoxicity can be reflected by the bioluminescence intensity.

*In vivo*, the extracted tumors were cut into small pieces and incubated with a digestive mix containing RPMI-1640 (Cat# 11875093,Gibco) with collagenase IV (50 μg/mL) for 1 h at 37°C. Tumor tissues were filtered to make single-cell suspensions and stained with the following antibodies: CD45 (APC, Clone 30-F11, Cat# 559864, BD; PerCP/Cyanine5.5, Clone 30-F11, Cat# 103129), CD3 (FITC; Clone 17A2, Cat# 100204 or PE; Clone17A2, Cat# 100206), CD8-PerCP/Cyanine5.5 (Clone 53-6.7, Cat# 551162, BD), NK1.1-FITC (Clone PK136, Cat# 11-5941-82, Ebioscience), IFN-γ-PE (Clone XMG1.2, Cat# 505807), TNF-α-PE (Clone MP6-XT22, Cat# 506306), GranzymeB-PE (Clone QA18A28, Cat# 396406), CD69-FITC(Clone H1.2F3, Cat# 104505), CD107a-FITC(Clone 1D4B, Cat# 121605), CD25-APC (Clone PC61, Cat# 557192, BD), CD4-FITC (Clone GK1.5, Cat# 100405), FOXP3-PE(Clone MF-14, Cat# 126404), PD1 (PE, Clone 29F.1A12, Cat# 135206; FITC, Clone 29F.1A12, Cat# 135213), LAG3-PE(Clone C9B7W, Cat# 125207), TIM3-PE(Clone B8.2C12, Cat# 134003). All antibodies were acquired from BioLegend unless stated otherwise. Flow cytometry analysis was performed on a FACSCaliber cytometer (BD). Data were analyzed by FlowJo software (Treestar, USA).

### Statistical analysis

All statistical analyses were performed using GraphPad Prism 8 (GraphPad Software Inc., CA, USA). All data are presented as the means ± standard deviation (SD)/standard error of mean (SEM). Student’s t-test or analysis of variance (ANOVA) was used to analyze differences. Survival curves were plotted according to the Kaplan-Meier method and the survival in different treatment groups was compared using a log-rank test. Significance was defined as P-values< 0.05.*p<0.05, **p<0.01, ***p<0.001, ****p<0.0001.

## Results

### Generation and characterization of the oncolytic recombinant vaccina viruses

**Figure 1:**
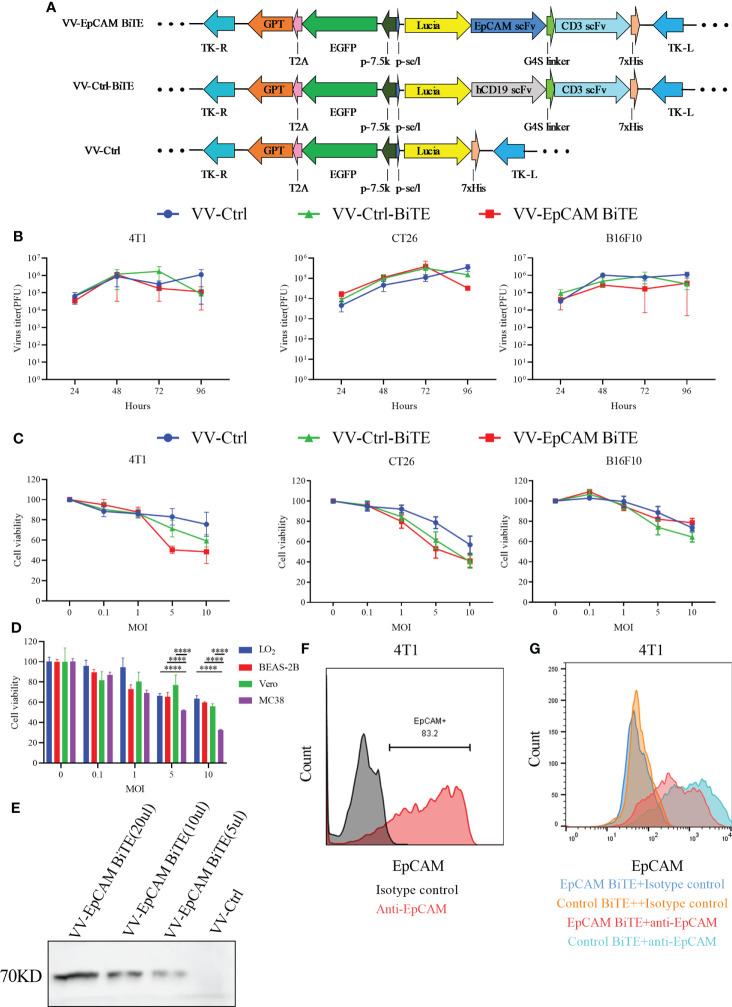
The construction and characterization of VV-EpCAM BiTE. **(A)** Schematic structure of recombinant vaccinia viruses. **(B)** Replication kinetics of VVs. 4T1, CT26, and B16F10 cells were infected with VV-Ctrl, VV-Ctrl-BiTE, or VV-EpCAM BiTE at an MOI of 1. Cells were harvested at 24, 48, 72, and 96 hours, and the virus titer was detected by titration assays in HEK 293 cells. All data are presented as means ± SEM (n=3). **(C)** Cytotoxic effects of VVs. 4T1, CT26 and B16F10 cells were infected with VV-Ctrl, VV-Ctrl-BiTE, or VV-EpCAM BiTE at the indicated MOIs. Cell viability was analyzed by MTT assay after 72 hours. All data are presented as means ± SEM (n=3). **(D)** Cytotoxic effects of VV-EpCAM BiTE. MC38, Vero, LO2 and BEAS-2B cells were infected with VV-EpCAM BiTE at the indicated MOIs. Cell viability was analyzed by MTT assay after 72 hours. All data are presented as means ± SD (n=3). **(E)** Expression and secretion of EpCAM BiTE from infected HEK293 cells. Anti-His-tag was used for western blotting. **(F)** Flow cytometry analysis of EpCAM expression on the surface of 4T1 cells. **(G)** Binding to 4T1 cells. 4T1 cells were incubated with supernatants harvested from VV-EpCAM BiTE- or VV-Ctrl-BiTE-infected HEK293 cells. Competitive inhibition of anti-EpCAM antibody binding by secreted EpCAM BiTE detected by flow cytometry.

EpCAM BiTE consisted of two scFV fragments linked with a flexible glycine-serine (GS) linker, and it targets mouse EpCAM and CD3. The control BiTE targeting human CD19 was used for nonspecific binding. Recombinant VVs encoding BiTEs were generated by inserting a p-se/l-derived transcription unit with foreign genes into the J2R (TK) locus of the VV genome. Meanwhile, an additional p-7.5k-derived transcription unit with a reporter gene (EGFP) and a screening gene (GPT) were also inserted into the TK locus. A recombinant VV expressing only EGFP (VV-Ctrl) was used as another control (**Figure 1A**). The TK of the parental VV was inactive after insertion of these foreign genes. The recombinant viruses were screened and purified in HEK293 cells. The removal of the parental VV was validated by PCR amplification of the target gene and the TK gene (data not shown).

We next evaluated the replication capacity of these recombinant VVs in cancer cells. The results of the TCID50 assay showed no significant difference in viral titer at a series of time points after VV-Ctrl, VV-Ctrl-BiTE, and VV-EpCAM BiTE infection, suggesting that engineering VVs do not affect viral productivity (**Figure 1B**).

To evaluate whether the insertion of exogenous genes would change the oncolytic ability of VVs, B16F10, CT26, and 4T1 cells were infected with VV-EpCAM BiTE, VV-Ctrl-BiTE and VV-Ctrl, respectively, and dose (MOI)-dependent oncolytic activities were observed for all VVs (**Figure 1C**). At MOI of 5 and 10, VV-EpCAM BiTE showed significantly greater tumor lysis of MC38 than normal cells (p<0.0001), including LO2, BEAS-2B and Vero (**Figure 1D**).

The secretion of EpCAM BiTE in supernatants harvested from VV-EpCAM BiTE-infected HEK293 cells was detected by western blot (**Figure 1E**). We then assessed EpCAM expression in 4T1 cells and found that 4T1 cells expressed high levels of EpCAM (**Figure 1F**). Then, we performed a competition binding assay of secreted EpCAM BiTE from VV-EpCAM BiTE-infected 4T1 cells and found that EpCAM BiTE reduced the fluorescence intensity of anti-EpCAM with the same binding domain of EpCAM BiTE on 4T1 cells (**Figure 1G**), indicating that the secreted EpCAM BiTE was effectively bound to EpCAM on the cell surface.

### Secreted EpCAM BiTE effectively induces T cells activation

**Figure 2:**
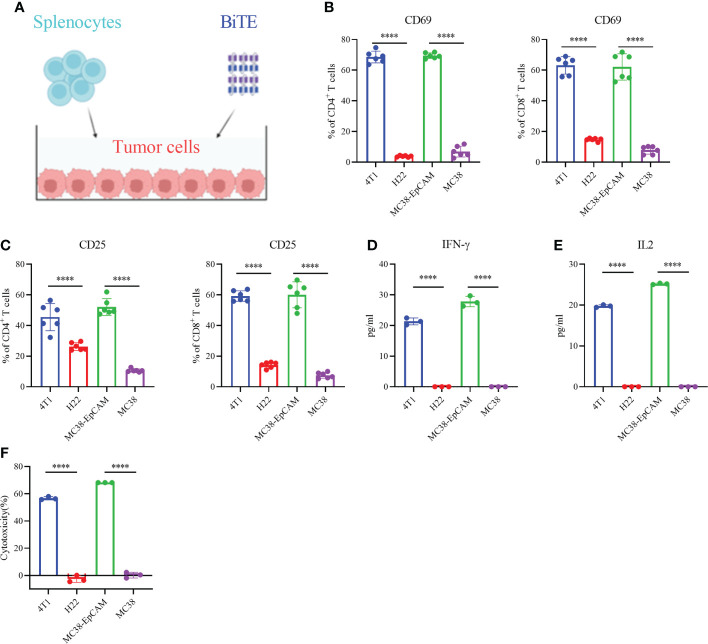
Secreted EpCAM BiTE induces naive T-cell activation and cytotoxicity of tumor cells. **(A)** Schematic diagram of the T-cell toxicity test. **(B, C)** Expression of CD69 and CD25 on the surface of CD4^+^ and CD8^+^ T cells. 4T1, H22, MC38-EpCAM and MC38 cells were cocultured with splenocytes at an effector to target (E:T) ratio of 10:1 in the presence of EpCAM BiTE. Cells were harvested at 48 hours and then assayed by flow cytometry. **(D, E)** BiTE-induced cytokine secretion. The secretion levels of IFN-γ and IL-2 were determined by enzyme-linked immunosorbent assay (ELISA). **(F)** T-cell cytotoxicity. 4T1, H22, MC38-EpCAM and MC38 cells were cocultured with murine splenocytes and EpCAM BiTE for 48 h for the luciferase assay, and the tumor cell lysis rate was calculated. ****p < 0.0001.

Then, we investigated whether EpCAM BiTE induces primary T cell activation and T cell-mediated cytotoxicity against tumors, the schematic is shown in **Figure 2A**. EpCAM BiTE specifically induced CD4^+^ and CD8^+^ T-cell activation, as indicated by a conspicuous increase in the expression of the activation markers CD69 and CD25 (**Figures 2B, C**). Notably, EpCAM BiTE induces CD4^+^ and CD8^+^ T-cell activation in the presence of EpCAM-positive tumor cells, like 4T1 and MC38-EpCAM, but not EpCAM-negative tumor cells, such as H22 and MC38. Furthermore, we assessed cytokine production in the coculture systems. Compared with H22 and MC38, EpCAM BiTE induced higher levels of IFNγ and IL-2 release in the presence of 4T1 and MC38-EpCAM (**Figure 2D, E**). Finally, we investigated the immunocytotoxicity of EpCAM BiTE and found that BiTE significantly enhanced the cytotoxicity of T cells against 4T1 and MC38-EpCAM cells compared to H22 and MC38 cells. (**Figure 2F**). Taken together, these results indicate that EpCAM BiTE can functionally engage T-cell cytotoxicity in the presence of EpCAM-positive target cells.

### VV-EpCAM BiTE exhibits a superior antitumor activity in EpCAM-expressing breast cancer

**Figure 3:**
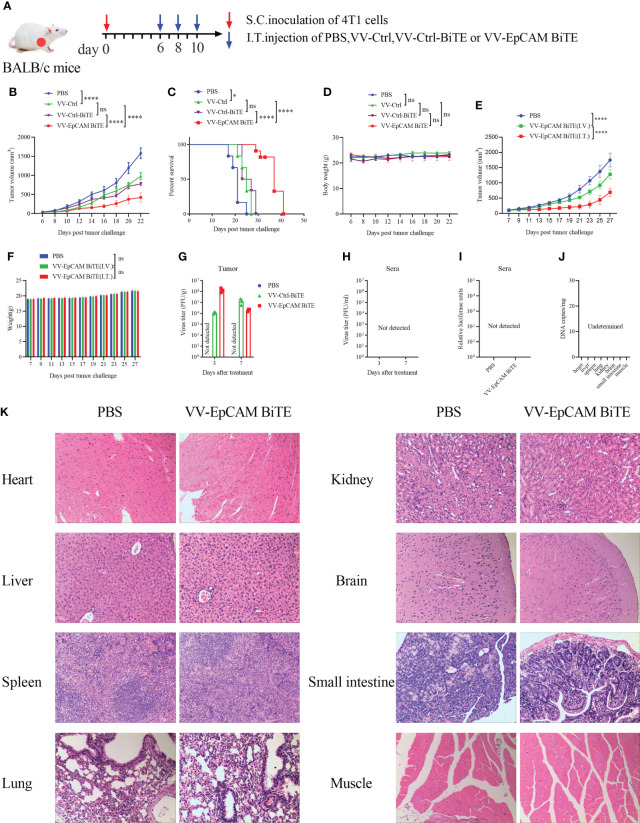
Antitumor effect of VV-EpCAM BiTE in the 4T1 model. **(A)** Experimental timeline for 4T1 models. BALB/c mice were subcutaneously inoculated with 2x10^5^ 4T1 cells. 6 days later, tumor-burdened mice were intratumorally injected with PBS or VVs at a dose of 1x10^7^ PFU per mouse 3 times. **(B)** The volumes of tumor were monitored by caliper measurement. Data are presented as means ± SEM. **(C)** Kaplan-Meier survival curves of 4T1-bearing mice. **(D)** Body weight of 4T1-bearing mice. **(E)** Tumor volume of mice injected VV-EpCAM BiTE. I.V, intravenous injection; I.T, intratumoral injection. Data are presented as means ± SD. **(F)** Body weight of mice injected with VV-EpCAM BiTE. **(G, H)** Replication of VVs in the tumor tissue and sera was detected by titration assays. **(I)** The EpCAM BiTE levels in blood. Sera were prepared and a luciferase assay was used to detect the levels of secreted EpCAM BiTE. **(J)** Replication of VV-EpCAM BiTE in mouse organs or tissues. **(K)** Hematoxylin and eosin (H&E) staining of heart, liver, spleen, lung, kidney, brain, small intestine and muscle tissues. 2 × 10^5^ 4T1 cells were inoculated in the right axil of BALB/c mice to establish a subcutaneous tumor model. When the tumor reached approximately 100 mm^3^, the mice were injected intratumorally with PBS or 1×10^7^ PFU VV-EpCAM BiTE. After 48h, the mice were sacrificed and the tissues were collected for HE staining. ns, no significant differences; *p < 0.05; ****p < 0.0001.

To assess the antitumor efficacy of recombinant VVs, 4T1 tumor-bearing mice received intratumoral injections of PBS, VV-Ctrl, VV-Ctrl-BiTE or VV-EpCAM BiTE (**Figure 3A**). VV-EpCAM BiTE significantly inhibited tumor growth (**Figure 3B**) and prolonged survival (**Figure 3C**). No significant toxicity was observed during the treatment as monitored by mouse body weight (**Figure 3D**). These data revealed that VV-EpCAM BiTE is superior to VV-Ctrl and VV-Ctrl-BiTE in reducing tumor burden and prolonging survival in breast cancer-bearing mice. Meanwhile, we also observed the efficacy of VV-EpCAM BiTE when injected intravenously and found that compared with PBS group, intravenous VV-EpCAM BiTE had a positive therapeutic effect with no significant change in body weight and no mouse mortality during treatment (**Figures 3E, F**). Not surprisingly, the tumor volumes of VV-EpCAM BiTE intratumourally injected mice were significantly smaller than those of VV-EpCAM BiTE intravenously injected mice (**Figure 3E**).

Viral replication *in vivo* was also monitored after intratumoral injection of VVs by viral titer in both tumor tissue and sera. Viruses were detected in tumor tissues on the third and seventh days after virus treatment, but not in sera from mice treated with VVs (**Figures 3G, H**). Compare to PBS, no circulating BiTE was detected in the blood of mice treated with VV-EpCAM BiTE, which was detected by luciferase activity (**Figure 3I**). 48h after intratumoral injection of VV-EpCAM BiTE, no virus was detected in organs or tissues including heart, liver, spleen, lung, kidney, brain, small intestine and muscle (**Figure 3J**). Consistently, no damage to these tissues was observed in VV-EpCAM BiTE treated mice (**Figure 3K**).

**Figure 4:**
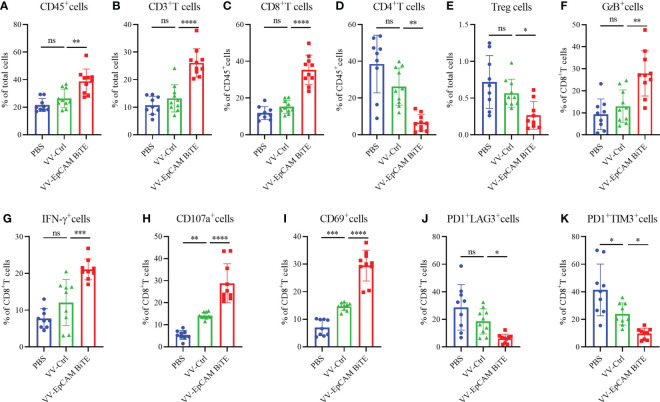
Immune cell infiltration of 4T1 model. **(A-K)** Preparation of single cell suspensions from digested tumor tissue as previously described in Materials and Methods. Flow cytometry analysis of the proportion of CD45^+^ cells **(A)**, CD3^+^ T cells **(B)**, CD8^+^ T cells **(C)**, CD4^+^ T cells **(D)**, Treg cells **(E)**, GzB^+^ cells **(F)**, IFN-γ^+^ cells **(G)**, CD107a^+^ cells **(H)**, CD69^+^ cells **(I)**, PD1^+^ LAG3^+^ cells **(J)**, and PD1^+^ TIM3^+^ cells **(K)**. Data are presented as the means ± SD (n = 9-10). ns, no significant differences; *p < 0.05; **p < 0.01;***p < 0.001; ****p < 0.0001.

Flow cytometry results showed a progressive increase in CD45^+^, CD3^+^ and CD8^+^ cells within the tumors of mice treated with VV-Ctrl or VV-EpCAM BiTE compared to mice treated with PBS. However, there was no statistical difference in CD45^+^, CD3^+^ and CD8^+^ cells between VV-Ctrl group and PBS group. Compared with VV-Ctrl group, VV-EpCAM BiTE treatment significantly increased the infiltration of CD45^+^, CD3^+^ and CD8^+^ cells (p<0.001, P<0.0001, P<0.0001) (**Figures 4A-C**). The proportion of CD4^+^ and Treg cells was comparable between VV-Ctrl and PBS group. Notably, the proportion of CD4^+^ and Treg cells was significantly lower in the VV-EpCAM BiTE treated-group compared to the VV-Ctrl treated-group. (**Figures 4D, E**). In addition, VV-EpCAM BiTE was found to more effectively activate CD8^+^ T cells than VV-Ctrl, as evidenced by increased expression of CD69 and CD107a on the cell surface and increased expression of intracellular IFN-γ and granzyme B (**Figures 4F–I**). At the same time, VV-EpCAM BiTE reduced the exhaustion of CD8^+^ T cells, which was mainly reflected in a reduction in the proportion of PD1^+^LAG3^+^ and PD1^+^TIM3^+^ cells in CD8^+^ T cells (**Figures 4J, K**). Taken together, VV-EpCAM BiTE displayed outstanding antitumor efficacy and a powerful ability to recruit and activate CD8^+^ T cells in a 4T1 breast model.

### VV-EpCAM BiTE and VV-Ctrl share similar antitumor effects in the EpCAM-negative carcinoma model

**Figure 5:**
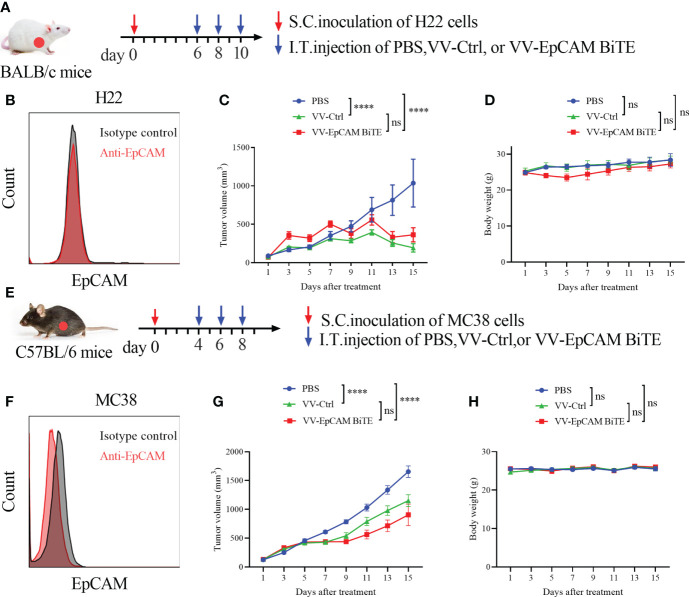
Antitumor effect of VV-EpCAM BiTE in the EpCAM-negative tumor model. **(A)** Experimental timeline for H22 models. BALB/c mice were subcutaneously inoculated with 2x10^6^ H22 cells. When the tumor size reached 100 mm^3^ (Day 6), VV-Ctrl, VV-EpCAM BiTE (1 × 10^7^ pfu) or PBS was injected intratumorally into the mice, and this was repeated on Days 8 and 10. **(B)** Flow cytometry analysis of EpCAM expression on the surface of H22 cells. **(C)** The volumes were monitored by caliper measurement once every other day. Data are presented as means ± SEM. **(D)** Body weight of H22-bearing mice. **(E)** Experimental timeline for MC38 models. C57BL/6J mice were subcutaneously inoculated with 2x10^6^ MC38 cells. When the tumor volume was approximately 100 mm^3^, randomized groups were treated with VVs (1 × 10^7^ pfu) or PBS. **(F)** Flow cytometry analysis of EpCAM expression on the surface of MC38 cells. **(G)** Caliper measurements were made on the MC38 tumors. **(H)** Body weight of MC38-bearing mice. ns, no significant differences; ****p < 0.0001.

We then assessed EpCAM expression in H22 and MC38 cells and found that both H22 and MC38 cells lacked EpCAM expression (**Figures 5B, F**). To verify our hypothesis, a model of H22 was established in BALB/c mice. The tumor-bearing mice were treated at 2-day intervals 3 times *via* intratumoral injection (**Figure 5A**). VV-Ctrl- and VV-EpCAM BiTE-treated tumors grew more slowly than those in the PBS group (P< 0.0001; P< 0.0001) (**Figure 5C**). Reassuringly, there was no significant difference in tumor volume between VV-Ctrl- and VV-EpCAM BiTE-treated mice (P > 0.05). In addition, no difference was observed in body weight among these groups (P > 0.05) (**Figure 5D**).

Next, we established an MC38 model, and tumor-bearing C57BL/6J mice were treated 3 times *via* intratumoral injection (**Figure 5E**). The tumor volumes of mice treated with VV-Ctrl and VV-EpCAM BiTE were significantly lower than those of mice treated with PBS (P< 0.0001; P< 0.0001) (**Figure 5G**). However, there was no significant difference in tumor volume between the VV-Ctrl and VV-EpCAM BiTE groups (P > 0.05). Additionally, no significant toxicity, as monitored by mouse body weight, was observed during the treatment (**Figure 5H**). These results collectively indicated that in mouse models where tumor cells expressed few of EpCAM, VV-EpCAM BiTE was unable to exert antitumor effects beyond VV-Ctrl.

### Antitumor activity of VV-EpCAM BiTE in the MC38-EpCAM cancer model

**Figure 6:**
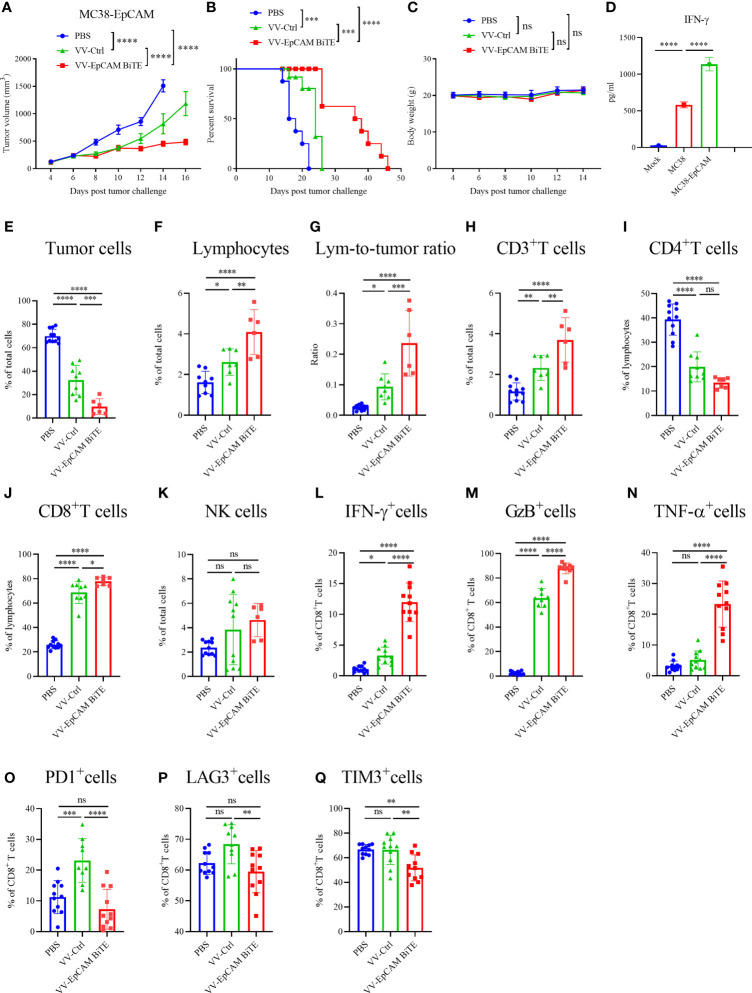
Antitumor effect of VV-EpCAM BiTE in the MC38-EpCAM model. **(A)** The volumes of MC38-EpCAM tumors were monitored by caliper measurement. Data are presented as means ± SEM. **(B)** Kaplan-Meier survival curves of MC38-EpCAM-bearing mice. **(C)** Body weight of MC38-EpCAM-bearing mice. **(D)** Expression levels of IFN-γ. After 1 x 10^7^pfu VV-EpCAM BiTE treatment of tumor-bearing mice for 48h, mice were sacrificed and splenocytes were prepared. Splenocytes were co-incubated with MC38 or MC38-EpCAM cells for 48h. The production of IFN-γ was measured by ELISA. **(E-Q)** Flow cytometry analysis of the proportion of tumor cells **(E)**, lymphocytes **(F)**, lymphocyte-to-tumor ratio **(G)**, CD3^+^ T cells **(H)**, CD4^+^ T cells **(I)**, CD8^+^ T cells **(J)**, NK cells **(K)**, IFN-γ^+^ CD8^+^ T cells **(L)**, GzB^+^ CD8^+^ T cells **(M)**, TNF-α^+^ CD8^+^ T cells **(N)**, PD1^+^ CD8^+^ T cells **(O)**, LAG3^+^ CD8^+^ T cells **(P)** and TIM3^+^ CD8^+^ T cells **(N)**. Data are presented as the means ± SD (n = 6-11). ns, no significant differences; *p < 0.05; **p < 0.01; ***p < 0.001; ****p < 0.0001.

To verify the specificity of EpCAM BiTE, we established the EpCAM-positive stable cell line MC38-EpCAM. Afterward, the expression of EpCAM was identified by flow cytometry (data not shown). Furthermore, we explored the antitumor effect of VV-EpCAM BiTE in mice with MC38-EpCAM cells. The treatment scheme is similar to **Figure 5E**. Administration of both VV-Ctrl and VV-EpCAM BiTE significantly inhibited tumor growth (**Figure 6A**) and prolonged survival (**Figure 6B**). In addition, VV-EpCAM BiTE showed more prominent effects on tumor suppression and survival than VV-Ctrl (**Figures 6A, B**). No significant difference was observed in body weight among the three groups (P > 0.05) (**Figure 6C**). MC38-EpCAM tumor-bearing mice were treated with VV-EpCAM BiTE, then splenocytes were isolated and co-incubated with MC38-EpCAM or MC38 cells. As a result, both MC38-EpCAM and MC38 cells elicited high levels of IFN-γ from splenocytes, in which MC38-EpCAM induced IFN-γ was even higher, indicating that the VV-EpCAM BiTE mediated immune responses in mice was specific to both MC38 tumor and EpCAM (**Figure 6D**). Collectively, these *in vivo* and *in vitro* data demonstrated that intratumoral injections with VV-EpCAM BiTE were able to provoke potent antitumor responses in EpCAM-expressing tumors.

Furthermore, we analyzed the infiltration of immune cells into the tumor after intratumoral injection of VV-EpCAM BiTE. VV treatment markedly reduced the proportion of tumor cells in the tumor tissue compared with PBS treatment. Of note, VV-EpCAM BiTE-treated mice showed a significantly lower proportion of tumor cells than VV-Ctrl-treated mice (P = 0.0002) (**Figure 6E**). VV-intratumoral injection significantly enhanced the tumor infiltration of total lymphocytes and CD3^+^ and CD8^+^ T cells. In addition, VV-EpCAM BiTE-treated mice exhibited higher infiltration of total lymphocytes and CD3^+^ and CD8^+^ T cells than VV-Ctrl-treated mice (p = 0.0070, p = 0.0048, p = 0.0129) (**Figures 6F, H, J**). Consistent with these results, the lymphocyte-to-tumor cell ratios of VV-Ctrl-treated mice and VV-EpCAM BiTE-treated mice were higher than that of PBS-treated mice (**Figure 6G**). Significant reduction in the proportion of CD4^+^ T cells after VV treatment compared to the PBS group (**Figure 6I**). VV injection did not noticeably enhance the composition of NK cells in the tumor tissue (**Figure 6K**).

Moreover, tumor-infiltrated CD8^+^ effector T cells were more efficiently activated by VV-EpCAM BiTE injection than by VV-Ctrl injection or PBS injection, as evidenced by the enhanced expression of IFN-γ, granzyme B and TNF-α (**Figures 6L–N**). The proportion of PD1^+^, LAG3^+^ and TIM3^+^ in CD8^+^ T cells of VV-EpCAM BiTE-treated mice was reduced to lower levels than in VV-Ctrl-treated mice. (**Figures 6O–Q**). These results are consistent with intratumoral immune cell infiltration in VV-EpCAM BiTE treated 4T1 tumor-bearing mice. Taken together, we proved that VV-EpCAM BiTE possessed the ability to reform the tumor microenvironment by enriching immune cell infiltration, activating tumor-infiltrating effector T cells and reducing CD8^+^ T-cell exhaustion.

## Discussion

Bispecific T-cell engagers (BiTEs) face several obstacles in solid tumors, including systemic toxicity and the immunosuppressive TME (27, 30). In this study, we combined oncolytic virotherapy and BiTE as a therapeutic agent and aimed to overcome some of the limitations of BiTEs by destroying tumor cells through direct oncolysis and specific activation of cytotoxic activity of T cells. Experimentally, we constructed a novel recombinant oncolytic vaccinia virus encoding an EpCAM BiTE (VV-EpCAM BiTE), which was able to efficiently replicate and lyse tumor cells *in vitro*, and efficiently activated intratumoral immune responses without exogenous stimulation resulting in improved antitumor outcomes in EpCAM positive malignancies.

We selected EpCAM to serve as a target antigen because EpCAM is a pancancer marker that is highly expressed on cancer cells and has been shown to be a marker for cancer-initiating stem cells (31–33). As an activator of the WNT pathway, EpCAM is thought to have a direct relationship with tumor progression (33). In breast and gallbladder cancers, high EpCAM expression is associated with poor prognosis (34). Its differential expression in normal and malignant tissues may provide a therapeutic strategy for treating various EpCAM^+^ epithelial cancers (35). Even so, solitomab, an EpCAM-CD3 BiTE, was still reported to damage healthy EpCAM^+^ tissues in phase I clinical trials (36, 37). Therefore, local administration *via* an oncolytic virus can avoid the side effects of BiTE (38). In our study, no significant toxicity was observed during the treatment as monitored by mouse body weight.

Several strains of vaccinia virus are currently available worldwide, such as the Lister, Wyeth, Copenhagen, Western Reserve (WR), New York City Board of Health (NYCBH), and Tiantan strains (TTV) (39). As reported, the WR strain is the most tumourolytic VV strain in animal models, and 10^6^ pfu of WR strain vaccinia virus can rapidly cause subcutaneous necrotic ulcers in rhesus monkeys with no systemic transmission of the virus (40). Our laboratory uses the WR strain and enhances the tumor-specific selection of vaccinia virus by deleting the thymidine kinase (TK) gene (14, 38). Vaccinia viruses lacking the TK gene have been shown to be injected intravenously or intraperitoneally and are carried into subcutaneous tumors where they replicate and cause an anti-tumor response (41).

BiTE has prominent therapeutic advantages as a specific tumor immunotherapy without the requirement of MHC-1 antigen presentation (42). When oncolytic viruses are used as a transgene platform to deliver BiTE, selective replication of oncolytic viruses in tumors locally can reduce the possibility of the “On target, off tumor” effect of BiTE (43). Fajardo et al. engineered an oncolytic adenovirus, ICOVIR-15K, to express a BiTE targeting EGFR (44). EGFR-BiTE-ICOVIR-15K was able to replicate and lyse tumor cells *in vitro*. In A549 and HCT116 xenograft models in immunodeficient SCID/beige mice, both intratumoral and intravenous injection significantly enhanced antitumor efficacy (44). In 2017, Freedman et al. constructed recombinant oncolytic adenovirus-encoded EpCAM-targeted BiTEs (EnAd-SA-EpCAMBiTE and EnAd-CMV-EpCAMBiTE) and validated their antitumor efficacies in patients with pleural effusions and ascites (45). This study demonstrates for the first time the efficacy of OV-BiTE in cancer biopsies. However, the lack of a three-dimensional structure is a drawback of this model, which hinders the study of the biodistribution of viruses and immune cells *in vivo* induced by OV-BiTE injections. Furthermore, the advantages of OV-BiTE over purified BiTE could not be illustrated in this liquid tumor model. Therefore, it would be valuable to investigate the antitumor efficacy of OV-BiTE in immunocompetent mice. Here, we investigated the antitumor effects of intratumoral injections of VVs in immunocompetent C57BL/6J and BALB/c mice. In the tumor with high EpCAM expression such as 4T1 and MC38-EpCAM models, VV-EpCAM BiTE exerted a more potent antitumor effect than VV-Ctrl. However, in the mice bearing tumors with low EpCAM expression, VV-EpCAM BiTE had similar efficacy to VV-Ctrl. These results confirm the specific antitumor potential of EpCAM BiTE. Additionally, we found that with intratumoral injection of VVs, virus copies were only detected locally in the tumor and no systemic transmission of the virus was detected. Also, no BiTE was detected in the circulating blood. Intravenous injection of VV-EpCAM BiTE significantly inhibited tumor growth in mice with no significant change in body weight compared to the control group, and no mice died during the treatment period. These results demonstrate the safety and efficacy of VV-EpCAM BiTE.

T cells play a key role in the destruction of tumors, where the durability and functionality of T cells determine the efficacy of immunotherapy (46). The OV-BiTE platform leverages the best features of both oncolytic virus and BiTE treatments while overcoming many of the barriers encountered with monotherapy. Oncolytic viruses increase the infiltration of immune cells into the tumor microenvironment (47), but the efficacy of oncolytic virus monotherapy for solid tumors is less satisfactory due to problems such as antiviral responses and lack of tumor specific immune responses (48). It is necessary to combine oncolytic viruses with other agents to improve their antitumor effects. BiTE could help overcome some limitations of viral therapy. The antitumor immune response of T cells depends mainly on the expression of MHC-I, which is downregulated in cancer cells (49). BiTEs, which are theoretically independent of MHC-I and the polyclonal mode of action, can nonselectively activate tumor-infiltrating T cells (including antiviral-specific T cells) and redirect them to cancer cells (50). At the same time, BiTE produced by infected cancer cells is locally enriched in the tumor, thus avoiding systemic toxicity and maximizing efficacy. EpCAM BiTE induced increased expression of CD25 and CD69 on the surface of CD4^+^ and CD8^+^ T cells and a significant increase in the number of IFN-γ-producing T cells. *In vivo*, the CD8^+^ T-cell infiltration of mice treated with VV-EpCAM BiTE was much higher than that of mice treated with PBS, indicating that VV-EpCAM BiTE has a distinguished capacity to recruit T cells, converting “cold” tumors into “hot” tumors. VV-EpCAM BiTE-treated mice showed more CD8^+^ T-cell infiltration than VV-Ctrl-treated mice. Moreover, these mice showed significantly higher levels of IFN-γ and granzyme B, as well as a lower proportion of PD1^+^, LAG3^+^ and TIM3^+^ in CD8^+^ T cells in the tumor tissue, indicating a higher level of CD8^+^ T-cell activation. Despite the superior antitumor performance of VV-EpCAM BiTE in the treatment of solid tumors, it did not completely cure the tumor-bearing mice. We hypothesized that activation of T cells by CD3 alone in a suppressive tumor environment would not be sufficient to eradicate tumor burdens and that the persistence of T cells is the key to ensure long-term tumor control (51). Additional genetic modification of VV-EpCAM BiTE with transgenes that encode costimulatory molecules or cytokinesis is a potential strategy to improve its antitumor efficacy.

In conclusion, our findings provide preclinical evidence for the therapeutic potential of VV-EpCAM BiTE. Our results demonstrate that BiTE-armored oncolytic VVs have the unique properties of inducing specific and redirected antitumor immune responses. This strategy has the potential to address key limitations in the application of oncolytic virotherapy and BiTE therapies in solid tumors, and our results support the value of further evaluation and development of this strategy.

## Data availability statement

The original contributions presented in the study are included in the article/supplementary material. Further inquiries can be directed to the corresponding authors.

## Ethics statement

The animal study was reviewed and approved by the Animal Care and Use Committee of the Medical School of Nanjing University.

## Author contributions

Conception and design: SZ, JD, JW. Conduction of the experiments: MW, ZC, PQ, YZ, LK, HG Analysis of the data: MW, SZ, JD, JW. Writing, review, and/or revision of the manuscript: MW, PQ, JD, JW. All authors contributed to the article and approved the submitted version.

## Funding

This work was supported by National Natural Science Foundation of China (81773255 and 81700037).

## Conflict of interest

The authors declare that the research was conducted in the absence of any commercial or financial relationships that could be construed as a potential conflict of interest.

## Publisher’s note

All claims expressed in this article are solely those of the authors and do not necessarily represent those of their affiliated organizations, or those of the publisher, the editors and the reviewers. Any product that may be evaluated in this article, or claim that may be made by its manufacturer, is not guaranteed or endorsed by the publisher.
